# Circadian Systems and Neural Control of Blood Pressure

**DOI:** 10.1007/s11906-026-01390-7

**Published:** 2026-09-16

**Authors:** Hiruni M. Aponso, Jennifer S. Pollock, Bryan K. Becker, David M. Pollock

**Affiliations:** Section of Cardio-Renal Physiology and Medicine, Division of Nephrology, Department of Medicine, University of Alabama at Birmingham, 1918 University Blvd, MCLM 511, Birmingham, AL 35233 USA

**Keywords:** Circadian rhythm, Autonomic function, Clock genes, Sympathetic nervous system

## Abstract

**Purpose of Review:**

Blood pressure (BP) follows a robust circadian rhythm and disruption of this rhythm is associated with hypertension and increased cardiovascular disease risk. This review discusses the current understanding of how circadian mechanisms regulate the neural control of BP, with a focus on autonomic brain circuits, local molecular clocks, and emerging neuroimmune mechanisms contributing to the regulation of BP.

**Recent Findings:**

Numerous studies show that autonomic control areas of the brainstem are important for circadian BP regulation. Regions such as the rostral ventrolateral medulla (RVLM), the nucleus tractus solitarius (NTS), and paraventricular nucleus of the hypothalamus (PVN) all contain intrinsic molecular clocks that play a role in sympathetic activity and baroreflex function. Emerging evidence also indicates that disruption of clock function in microglia within the RVLM acts as a potential mechanism that contributes to neuroinflammation, sympathetic overactivity, and non-dipping hypertension. The growing interest in chronotherapy, renal denervation, and ambulatory BP monitoring emphasizes the clinical importance of circadian BP regulation.

**Summary:**

Circadian regulation of BP is comprised of coordinated interactions between the SCN, autonomic neural circuits, and local molecular clocks. Disruptions in any of these mechanisms lead to abnormalities in BP rhythms increasing the risk of hypertension. Despite recent advances, many questions remain regarding the relative contributions of central and peripheral clocks, the causal role of circadian disruption in hypertension, and the mechanisms underlying sex differences. Addressing these gaps may identify new approaches for preventing and treating hypertension by targeting circadian regulation of cardiovascular function.

## Introduction

Despite numerous antihypertensive therapies available, blood pressure (BP) control remains poor with fewer than half of treated patients reaching the recommended target BP values [1]. These limitations stress the need for a better understanding of the mechanisms regulating BP that contribute to the development of hypertension.

**Fig. 1:**
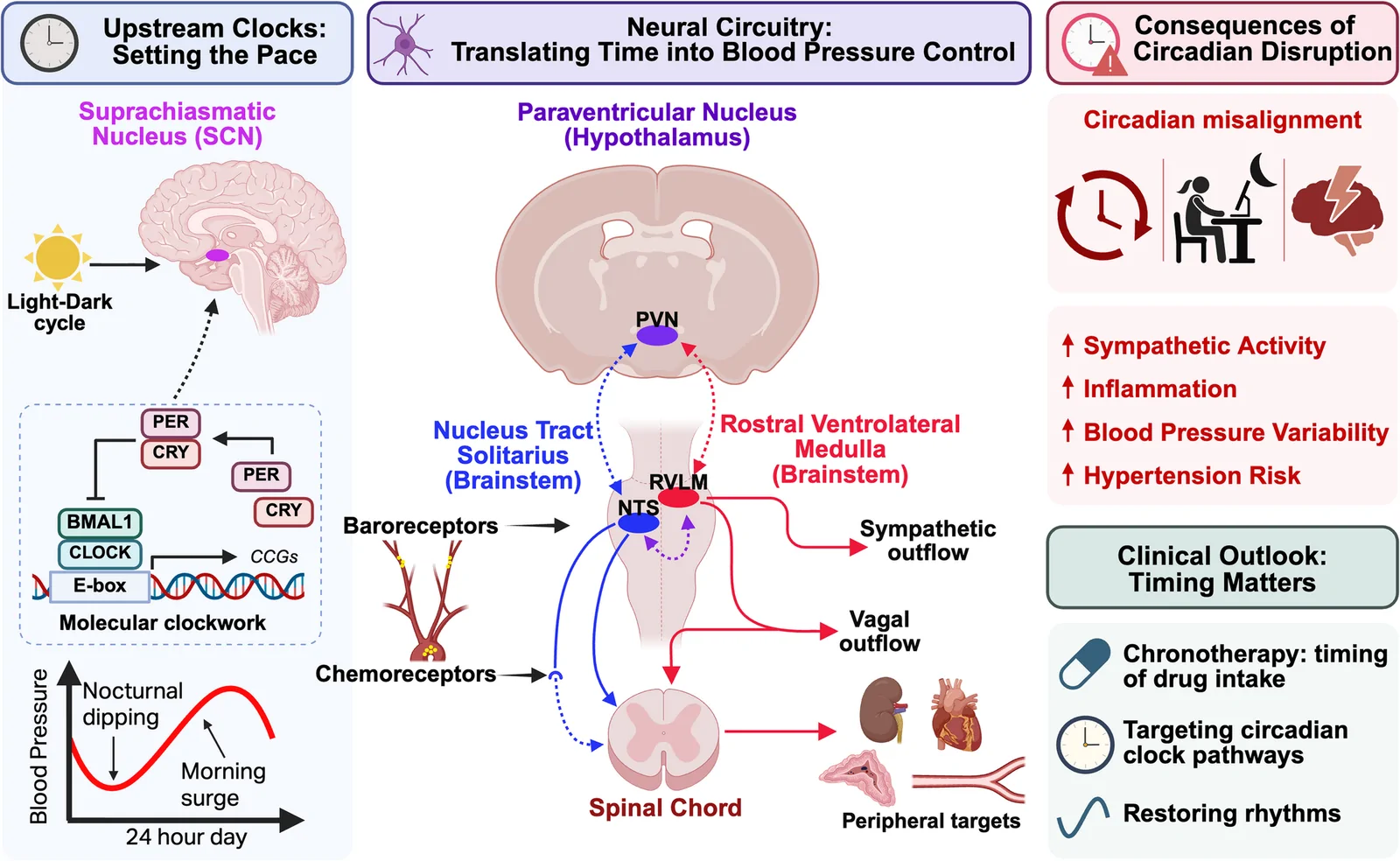
Summary of circadian control of autonomic function including the clinical relevance. Created in BioRender. Aponso, H.M. (2026) https://BioRender.com/t3ys8g3

In healthy individuals, BP follows a robust circadian rhythm in which BP is reduced by 10–20% during sleep, and upon waking there is a rapid increase in BP. These patterns are referred to as nocturnal dipping and the morning surge, respectively (Fig. 1) [2, 3]. Among hypertensive individuals, disrupted BP rhythms are a common characteristic. One of the most prevalent instances of BP rhythm disruption is a non-dipping phenotype. Non-dipping is defined as a nocturnal BP decline of less than 10%, affecting 30–50% of hypertensive patients [2, 4]. Non-dipping individuals have a greater risk of stroke, chronic kidney disease, and all-cause mortality [3, 5]. However, the clinical significance of dipping status should be considered alongside absolute daytime and nighttime BP levels. Dipping is expressed as a relative change between daytime and nighttime BP. An individual may have a normal dipping percentage despite elevated nighttime BP, or have a non-dipping pattern without an elevated nighttime BP. Therefore, both nocturnal BP levels and the magnitude of the day night BP change may provide important information about cardiovascular risk [3, 6]. Accordingly, throughout this review, disrupted BP rhythms are considered in the context of both BP rhythmicity and the absolute BP levels at different times of the day.

The circadian timing system is composed of a central clock found in the suprachiasmatic nucleus (SCN) (Fig. 1). The SCN synchronizes physiological processes and peripheral clocks to external timing cues such as light [7]. At the molecular level, circadian rhythms are created through transcriptional-translational feedback loops, often referred to as the TTFL (Fig. 1). On the positive arm of the TTFL are BMAL1 and CLOCK, which heterodimerize to E-box elements in promoter regions of many genes including the negative arm, PER1/PER2/PER3, and CRY1/CRY2 (Fig. 1) [8]. Experimental loss of BMAL1 disrupts BP rhythms and modifies cardiovascular responses to circadian, dietary, and environmental stressors, emphasizing the critical nature of the endogenous molecular clock in cardiovascular regulation [9–12]. The SCN communicates with hypothalamic and brainstem autonomic nuclei to coordinate timing of sympathetic activity, parasympathetic tone, and neuroendocrine responses (Fig. 1) [13]. These autonomic centers also have intrinsic molecular clocks that respond to local signals such as neuroinflammation, metabolic status, and synaptic activity [14]. These rhythmic SCN signals and local clock mechanisms within autonomic centers coordinate the central outputs that regulate daily rhythms of BP [7, 8].

In this review, we focus on the current understanding of how circadian mechanisms regulate the neural control of BP [13, 15–17]. We first introduce the neural circuits involved in BP regulation. The reader is referred to other more general recent reviews focused on autonomic control of BP and hypertension risk [13, 15–17]. We then discuss how the circadian system influences autonomic activity, baroreflex function, and cardiovascular physiological rhythms. Emphasis is placed on molecular clock mechanisms within autonomic brainstem nuclei and discussions of the emerging evidence linking clock dysfunction to neuroinflammation and sympathetic overactivity. Finally, we discuss the clinical implications of these findings for the management of hypertension.

## Neural Control of Blood Pressure

### Brainstem Circuits

The rostral ventrolateral medulla (RVLM) is a key brainstem region involved in regulating sympathetic nerve activity and BP control. Pre-sympathetic neurons of the RVLM activate sympathetic preganglionic neurons in the spinal cord which then regulate sympathetic outflow to peripheral organs (Fig. 1) [13, 18]. An increase in RVLM neuronal activity leads to an increase in sympathetic nerve activity and thereby causes vasoconstriction, increased heart rate, and increased BP. On the other hand, inhibition of RVLM neurons causes mild hypotension, which emphasizes their role in maintaining basal sympathetic tone [19].

The RVLM integrates information from multiple upstream autonomic and cardiovascular control centers, including the paraventricular nucleus (PVN), nucleus tractus solitarius (NTS), and caudal ventrolateral medulla (CVLM) (Fig. 1) [13, 20]. These regions send signals to the RVLM related to baroreceptor activity, chemoreception and stress responses. Studies show that RVLM neurons have distinct neuronal populations that respond to different afferent inputs and project to specific targets [19, 21]. This network allows the RVLM to coordinate regional sympathetic outflow as well as fine tune cardiovascular function in response to changes in physiological conditions.

The NTS is the primary site in the brainstem that processes cardiovascular sensory input. The NTS directly receives signals from arterial baroreceptors through the glossopharyngeal and vagus nerves [20, 22]. When BP rises, increased baroreceptor activity stimulates NTS neurons, which ultimately inhibit RVLM pre-sympathetic neurons. This results in a decrease in sympathetic outflow to the vasculature and lowers BP which helps to maintain cardiovascular homeostasis [20]. Similar to the RVLM, the NTS also has distinct neuronal populations that contribute differently to cardiovascular regulation depending on their downstream targets [20]. These findings demonstrate that the NTS functions as an integrative center that helps coordinate multiple cardiovascular pathways. Additionally, studies have demonstrated time-of-day differences in neuronal activity and glutamatergic signaling within the NTS which may contribute to circadian regulation of BP [23].

### Hypothalamic Circuits

The PVN is a major autonomic center involved in regulating sympathetic outflow, neuroendocrine signaling, and BP [13, 15, 16, 19, 24]. Parvocellular neurons of the PVN project to the RVLM and spinal sympathetic preganglionic neurons to cause an increase in sympathetic nerve activity and contribute to cardiovascular regulation (Fig. 1). Increased activity of PVN neurons has been linked to several forms of experimental hypertension, including salt-sensitive, stress-induced, and renovascular hypertension [25–29].

In addition to its role in autonomic regulation, the PVN also plays a role in fluid and electrolyte homeostasis by producing vasopressin and oxytocin. Through connections to circumventricular organs and other autonomic nuclei, the PVN integrates information regarding circulating hormones, osmotic status, and cardiovascular function. The PVN also receives projections from the SCN, which allows circadian signals to have an influence on both autonomic and neuroendocrine pathways involved in BP regulation [13, 24]. As a result, the PVN is well positioned to coordinate circadian, hormonal, and cardiovascular signals that contribute to daily rhythms in BP.

The dorsomedial hypothalamus (DMH) is important in regulating the activity of the sympathetic nervous system and has been implicated in the control of stress responses, metabolism, and cardiovascular function [30]. DMH neurons project to the RVLM and other autonomic brain regions, and activation of these neurons causes an increase in sympathetic nerve activity, heart rate, and BP [30, 31]. The DMH also receives input from the SCN and exhibits circadian rhythms in neuronal activity which suggest that it may transmit circadian signals to connecting autonomic circuits [32, 33]. Although the DMH is well positioned to control circadian rhythms in sympathetic activity and BP, the specific neural pathways have yet to be clearly established.

### Forebrain and Limbic Structures

The subfornical organ (SFO) is a circumventricular organ that lies outside the blood-brain barrier and can therefore detect circulating hormones and other humoral signals, including angiotensin II and changes in osmolality [13]. Through projections to the PVN and other hypothalamic nuclei, the SFO influences both sympathetic activity and vasopressin release. Importantly, as a major site of central angiotensin II signaling in the brain, the SFO provides an important link between the renin-angiotensin system (RAS) and neural regulation of BP [13, 34].

The supraoptic nucleus (SON) is a major center for vasopressin and oxytocin production in the hypothalamus [24]. SON neurons respond to changes in osmotic status and receive input from other forebrain regions involved in fluid balance regulation. Because vasopressin secretion exhibits daily rhythms and contributes to fluid homeostasis and vascular function, it is likely that the SON plays a role in circadian regulation of BP [13, 24, 35, 36].

## Circadian Control of Autonomic System Activity

### Daily Rhythms in Sympathetic Nervous System Activity

The autonomic nervous system (ANS) is a central regulator of blood pressure. The ANS is composed of two arms, the sympathetic and parasympathetic nervous system. Activation of the sympathetic nervous system increases heart rate, vascular resistance, and renin release, whereas activation of the parasympathetic arm slows heart rate. To assess autonomic nerve activity, indirect measurements are made such as heart rate variability (HRV), blood pressure variability (BPV), baroreflex sensitivity (BRS), and circulating catecholamines. HRV analysis measures the variation in time between heart beats. Activation of the sympathetic system lowers HRV, and activation of the parasympathetic system increases HRV. Thus, this analysis gives us insight into the balance between the two arms of the ANS. These measurements allow us to see how changes in autonomic balance across the day contribute to circadian regulation of BP.

The sympathetic nervous system exhibits robust circadian variation activity, with higher activity during the active phase and lower activity during the rest phase [11, 37]. These rhythms play a significant role in daily BP rhythms. In humans, HRV analyses indicate a greater influence of the sympathetic arm during waking hours while the parasympathetic arm is more active during sleep, consistent with the normal pattern of daytime BP elevation and nocturnal dipping [11, 38].

Experimental studies show that both environmental and behavioral cues can modify autonomic rhythms. In a study by Hou et al., changing the time of feeding in global *Bmal1* knockout mice shifted the timing of sympathetic activity and BP rhythms [11, 39]. This demonstrates that peripheral cues interact with circadian timing mechanisms to influence cardiovascular regulation [11]. This highlights the importance sympathetic nervous system rhythms in coordinating daily changes in BP and raises the possibility that circadian disruption contributes to abnormal cardiovascular regulation.

### Morning Surge Mechanisms

The morning blood pressure surge (MBPS) is a critical period in which there is an increased risk of cardiovascular events. During these hours, there are increased incidences of myocardial infarction, sudden cardiac death, and stroke [40–44]. This surge results from multiple physiological processes, such as circadian driven increases in sympathetic activity, catecholamine and cortisol release, and behavioral changes such as awakening, postural shifts, and physical activity [40, 41, 45, 46].

Environmental and behavioral factors can further increase MBPS. For example, being in a cold environment in the morning increases sympathetic activity and BP in healthy adults, suggesting that ambient temperature influences the magnitude of the surge [41]. On the other hand, when circadian rhythms are misaligned due to changes in sleep timing or jet lag, there is an exaggerated MBPS. This is due to disturbances of autonomic balance and behavioral timing [47].

One of the key contributors to MBPS is the postural change from supine to upright position after waking up. This shift causes a gravitational pooling of blood, activation of baroreflex pathways, and compensatory increases in sympathetic outflow and heart rate. When there are abnormalities in baroreflex function or sympathetic reactivity, there is a greater increase in BP [38, 48]. Consistent with this, hypertensive individuals with heightened MBPS show reduced baroreflex sensitivity and altered autonomic balance, providing evidence for their role in excessive morning BP elevation [38]. These findings show that the morning surge in BP is sensitive to both environmental and circadian influences.

### Circadian Modulation of Baroreflex Sensitivity

Baroreflex sensitivity (BRS) is defined as the change in heart rate or sympathetic activity in response to a given change in BP. This reflex is important for buffering the short-term fluctuations in BP and for maintaining cardiovascular stability. BRS shows a clear circadian rhythm and plays an important role in regulating BP throughout the day [22, 49, 50]. In healthy individuals, BRS is higher during the inactive phase and lower during the active phase which inversely correlates with the typical rhythms of BP. When BRS is reduced during the active phase, it contributes to increases in BP levels and BP variability [49].

In a study by Visniauskas et al., angiotensin II (AngII)-induced hypertension in mice caused disruption of both BP rhythms and baroreflex activity [50]. They observed a blunting of normal daily rhythms in BP, heart rate, and activity, as well as a sex-specific reduction of baroreflex function. Only male mice exhibited reduced BRS in response to AngII [50]. These findings suggest that hypertension not only increases BP but also disrupts the normal circadian rhythms of autonomic reflexes which help stabilize cardiovascular function.

Circadian disruption itself also impairs baroreflex processing [4, 12]. Repeated 6-hour phase advances in light for 22 days reduce glutamatergic signaling within the NTS and decrease expression of glutamate receptor subunits [4]. These responses lead to an increase in BP and support the concept that circadian misalignment reduces BRS [4]. Additionally, peripheral modulators of baroreflex function also show circadian and sex-dependent effects. Natriuretic peptides such as atrial natriuretic peptide (ANP) and brain natriuretic peptide (BNP) enhance BRS in a sex-dependent manner. In females, there is a stronger effect that is likely due to a higher expression of natriuretic peptide receptors in the baroreceptor afferent pathways and brainstem nuclei [22]. These sex differences may contribute to variability in circadian BP regulation across sexes.

## Molecular Clocks Within Neural Circuits

### Clock Gene Expression in Autonomic Nuclei

The RVLM expresses core clock genes, including BMAL1, CLOCK, PER, and CRY, and exhibits circadian variation in neuronal activity that aligns with daily BP rhythms [14].

The NTS shows circadian variation in glutamatergic signaling which contributes to the daily regulation of autonomic function and baroreflex activity [23]. Under normal conditions, glutamatergic synaptic transmission within the NTS changes throughout the circadian cycle. This suggests that excitatory processing in the NTS is under the control of the circadian clock [23]. Buijs et al. provided evidence that the SCN receives direct input from the NTS, enabling it to respond to changes in BP. This suggests a feedback mechanism for BP regulation that is linked to the central circadian clock [51]. Together, these findings suggest that circadian disruption could impact processing of baroreceptor input, although whether glutamatergic signaling contributes to hypertension remains unclear.

The PVN also expresses clock genes and exhibits circadian rhythms in neuronal activity [13, 24]. Because the PVN receives direct input from the SCN, both central circadian signals and local molecular clocks likely play a significant role in the diurnal variation of PVN output. However, the specific clock components in pre-sympathetic neuron populations of the PVN remain unclear. Further studies are needed to define how local clock mechanisms within the PVN shape daily rhythms in BP regulation.

### Clock Genes and Neuronal Excitability

Clock genes regulate neuronal excitability by controlling the transcription of ion channels. BMAL1 regulates the expression patterns of voltage-gated sodium, potassium, and calcium channels which helps control daily rhythms in neuronal excitability within the SCN [8]. Beyond the SCN, circadian mechanisms that regulate electrophysiological properties have been observed across multiple brain regions [52]. This highlights a conserved role of the molecular clock in regulating neuronal function [52]. However, less is known about whether similar clock dependent mechanisms directly contribute to the regulation of autonomic circuits involved in BP regulation. Given that the RVLM, PVN, and NTS all express core circadian clock genes, local regulation of ion channel expression could generate time-of-day differences in neuronal excitability and sympathetic output. However, direct experimental evidence linking molecular clock function to intrinsic excitability within these regions remain limited.

### Glial and Neuroimmune Contributions

Neuroinflammation within autonomic brain regions has recently been recognized as a factor in the development of hypertension, and emerging evidence indicates that the related inflammatory signaling is regulated in a circadian manner [14, 24]. Microglia exhibit intrinsic circadian rhythms in gene expression, morphology, and cytokine release, which helps to maintain diurnal variation in immune surveillance and inflammatory tone [53–55].

Recent evidence has revealed that clock dysfunction in immune cells within the RVLM may contribute to circadian BP disruption in hypertension [14]. In a Sprague Dawley rat model of stress-induced hypertension by repeated electric foot shock and noise exposure, Zhang et al. showed that RVLM microglia have increased expression of the microglia proinflammatory activation marker, CD86 during the active phase, and dampened CLOCK rhythms [14]. This led to a loss of normal cytokine rhythmicity and a sustained pro-inflammatory state, indicated by an elevation in TNF-α and IL-1β [14]. This was in parallel with increased oxidative stress, impaired sirtuin 1 (SIRT1) signaling, and activation of high mobility group box-1 (HMGB1)-mediated inflammatory pathways. After restoring microglial clock function using SIRT1-enriched extracellular vesicles, they were able to normalize circadian gene expression, cytokine rhythms, and reversed the non-dipping phenotype [14]. These findings support a causal role of microglial clock dysfunction in RVLM-driven sympathetic overactivity and indicates that reprogramming local clock machinery can reverse hypertensive phenotypes [14].

Microglial activation is regulated by extracellular signaling pathways that also exhibit circadian variation. Under control physiological conditions, microglia maintain a homeostatic phenotype during the rest phase [14]. However, circadian disruption or chronic stress shifted microglial toward a pro-inflammatory state [14]. Activation of microglia increases autonomic output by increasing glutamatergic transmission and reducing inhibitory signals within sympathetic control centers [14]. One pathway that may contribute to this response is purinergic signaling through microglial P2 × 7 receptors. Extracellular ATP activates P2 × 7 receptors to promote microglial inflammatory signaling [24]. Because extracellular ATP levels in key circadian regions, such as the SCN, also exhibit daily variation, purinergic signaling might provide a link between circadian timing and neuroimmune activation, but this has yet to be fully established [24]. Together, these pathways suggest that microglia act as an interface between circadian timing mechanisms and neuroinflammatory control of sympathetic activity.

## Translational Implications

### Timing of Antihypertensive Medication

Chronotherapy is where antihypertensive medications are given in a timely manner to align with biological rhythms to improve efficacy and/or reduce dosing [7, 56]. The potential clinical application for chronotherapy for hypertension has been reviewed extensively by Bowles et al., (2018) who highlighted the importance of considering both daytime and nighttime BP regulation when determining medication timing. The circadian regulation of BP, combined with the increased cardiovascular risk associated with nocturnal hypertension, provides a strong rationale for chronotherapy to optimize BP control. Early clinical studies have suggested that taking medications before bed may improve nocturnal BP control and reduce cardiovascular disease risk [57–59]. However, these findings were not confirmed in larger randomized trials such as the TIME study where they found no significant difference between morning and evening dosing on cardiovascular outcomes [60]. These conflicting results suggest that there isn’t a single optimal time for all patients. Instead, treatment may need to be more personalized based on factors such as medication class, BP dipping status, and circadian characteristics.

Individual chronotypes have recently been emerging as a consideration in timing of medication intake. Chronotypes describe an individual’s natural sleep and wake time (commonly referred to as “early birds” or “night owls”). Recent studies analyzing the TIME study have shown that chronotype changes the relationship between medication timing and cardiovascular outcomes [61, 62]. Specifically, later chronotypes appeared to have more favorable cardiovascular outcomes when antihypertensive medications were taken in the evening, whereas earlier chronotypes appeared to benefit more from morning dosing [61, 62]. These findings support a shift towards more personalized chronotherapy [61]. Importantly, these findings do not establish a universal recommendation for timing of medication. Rather, they suggest that timing of medication may be considered in relation to an individual’s sleep-wake pattern and BP profile. This approach aims to consider antihypertensive treatment timing in relation to an individual’s sleep-wake pattern and BP rhythm characteristics, rather than relying only on fixed clock times.

An important consideration is that chronotype and socially imposed sleep timing are not always aligned. For example, a person with a late chronotype who needs to wake up early for work may experience a mismatch between their preferred biological timing and their required sleep-wake schedule. In such cases, clock time alone may not accurately reflect circadian phase. As such, medication timing based solely on a fixed bedtime would not be optimal. This highlights the need for studies that integrate chronotype, habitual sleep timing, occupational/social schedules, and individual BP rhythms when determining optimal timing of antihypertensive therapy. At present, chronotype-based dosing remains an emerging approach rather than a universal clinical recommendation.

### Renal Denervation and Circadian Blood Pressure Rhythms

Renal denervation (RDx) uses a catheter to ablate renal sympathetic nerves and is an approved therapy for resistant hypertension [63]. This method directly reduces sympathetic signaling to and from the kidney. Clinical trials have shown that RDx causes a sustained reduction in overall BP, which supports the idea that renal sympathetic nerves are important in the development and maintenance of hypertension. In addition to reducing overall BP, there has been recent clinical evidence suggesting that renal denervation may also improve disrupted BP rhythms [64]. Some reports have shown improvements in nocturnal BP, restoration of dipping status, and reductions in MBPS following RDx [65]. However, other studies found little to no effect on circadian BP rhythms despite reductions in overall BP [65]. Together, these findings suggest that sympathetic overactivity may contribute to disruptions in BP rhythmicity, but the effects of RDx on circadian BP regulation are not completely understood.

Preclinical studies have provided mechanistic insight into how renal sympathetic nerves may influence circadian BP regulation. In a rat model of metabolic syndrome, renal denervation normalized blood pressure rhythms and improved cardiovascular injury [66]. Similarly, renal denervation reduced BP and improved BP variation particularly in the rest phase in the spontaneously hypertensive rat [64]. These improvements were accompanied by reduced norepinephrine levels, normalization of RAS rhythmicity, and restoration of circadian gene expression in the kidney [64]. These findings suggest that renal sympathetic signaling contributes not only to increased BP, but also to the loss of BP rhythmicity in hypertension. The mechanisms underlying the effects of sympathetic nerve activity on BP likely involve both reduced sympathetic signaling to the kidney, and changes in afferent feedback to central autonomic circuits. These factors together may help to re-establish the coordination of the peripheral and central circadian clocks. While these findings are promising, further clinical studies are needed to determine whether renal denervation has specific effects on BP rhythmicity in humans.

### Neuromodulation of Central Autonomic Circuits

Neuromodulation represents another potential therapeutic approach. Baroreflex activation therapy (BAT), which electrically stimulates the carotid sinus baroreceptors, can reduce sympathetic activity and lower BP in patients with resistant hypertension [67]. BAT may also influence BP rhythmicity. In a study of patients with resistant hypertension and impaired nocturnal dipping, increasing BAT stimulation specifically during the nighttime increased systolic BP dipping and improved overall dipping patterns [68]. However, in this study, 24-hour, daytime, and nighttime BP did not significantly change [68]. These findings suggest that autonomic neuromodulation may influence BP rhythmicity in addition to lowering mean BP.

Non-invasive approaches to modulate autonomic pathways include transcutaneous auricular or vagal stimulation. These methods have shown modest reductions in BP and heart rate in clinical studies, but substantial heterogeneity still exists among these studies [69]. A recent sham-controlled trial was designed to directly test transcutaneous autonomic neuromodulation in patients with uncontrolled hypertension where they measured BPV, 24-hour BP, HRV, sleep, and autonomic function [70]. Thus, non-invasive neuromodulation is an emerging therapeutic avenue that may be partially relevant to hypertension characterized by excessive sympathetic activity. However, whether these approaches can specifically restore disrupted circadian BP rhythms, and whether their effects depend on the timing of stimulation, remains unknown. Future studies should therefore examine whether neuromodulation targeted to specific circadian phases can improve nocturnal BP, restore normal dipping, or reduce exaggerated MBPS.

### Ambulatory Blood Pressure Monitoring

Ambulatory blood pressure monitoring (ABPM) is one of the primary clinical tools for assessing 24-hour BP rhythms [71]. In contrast to in office measurements, ABPM has the diagnostic advantage of providing a more reliable assessment of nocturnal hypertension and exaggerated morning surge in BP [3]. Because these phenotypes likely reflect changes in autonomic regulation, baroreflex function, and circadian timing mechanisms, ABPM has the potential to link experimental studies of neurocircadian BP control with clinical hypertension.

The use of ABPM also allows for circadian phenotyping that can help identify high-risk BP patterns even before sustained hypertension develops. For example, disrupted BP rhythms and exaggerated MBPS have been shown to predict future hypertension in individuals with normal but elevated BP [3, 6, 72]. Similarly, elevated MBPS has been associated with an increased risk of arrhythmias, which highlights its broader cardiovascular relevance [40]. Another benefit of using ABPM is the ability to evaluate the influence of behavioral and environmental factors on BP. A recent study by Theodorakopoulou et al. showed that treatment timing in hemodialysis patients altered BP rhythms [73]. When dialysis was performed in the evening, there were more favorable dipping patterns and reduced morning surge compared to when dialysis was completed in the morning. These findings reinforce the importance of timing of treatment. Home dialysis also offers an opportunity to individualize treatment timing, however studies examining the effects on BP rhythmicity remain limited.

## Knowledge Gaps

### Are Circadian Disruptions a Causal Factor in Neurogenic Hypertension?

Although circadian disruption is strongly associated with hypertension, a clear causal relationship has not been completely defined [2]. In brief, epidemiological studies have consistently linked shift work, social jetlag, and sleep disorders with an increased risk of developing hypertension. Similarly, animal models of circadian disruption indicate increases in BP and loss of normal BP rhythmicity [12, 47]. However, key mechanistic and translational questions remain unclear.

A central issue is whether circadian disruption is a primary driver of hypertension or instead acts to exacerbate pre-existing susceptibility. Longitudinal studies with early circadian stress exposure through the development of hypertension are still needed to determine causality. It is also unclear how circadian disruption interacts with established risk factors such as diet, stress, and genetic predisposition. Although Bmal1 knockout models demonstrate that disruption of the endogenous molecular clock disrupts cardiovascular responses to circadian stress and dietary challenges [9–12], it is still unclear on how the interaction of these pathways may increase the risk of hypertension in otherwise healthy individuals. Defining these interactions will be important for understanding why only a subset of individuals exposed to circadian disruption develop sustained hypertension.

Mechanistically, the relative contribution of central versus peripheral clocks is still not known. Although there is evidence that both SCN driven neural pathways and peripheral clocks in the kidney, vasculature, and adrenal glands are important for BP regulation, it is unclear whether these specific tissue clocks individually could be primary drivers of hypertension or if their misalignment combines to increase risk.

Finally, it remains unclear whether restoring circadian alignment can reverse or improve hypertension in a clinical setting. Animal studies show that restoration of microglial clock function in the RVLM reverses hypertension [14]. Furthermore, restricting feeding to the optimal time of day re-establishes circadian rhythmicity in a range of models such as in mouse studies of diet-induced obesity [74]. However, it is currently unknown whether broader interventions such as light therapy, sleep scheduling, or time restricted eating can meaningfully restore BP rhythms in hypertensive patients.

### Sex Differences in Neural Control of Circadian Blood Pressure

Recent findings show sex differences in circadian blood pressure regulation, autonomic control, and responses to circadian disruption. However, the underlying mechanisms are not well defined [12, 22, 37, 50]. These studies suggest that males and females may have distinct neuro-circadian pathways that contribute to the development of hypertension. Sex differences are also evident in baroreflex and neurohumoral regulation. Natriuretic peptides have a greater effect on BRS in females, and this is associated with an increase in receptor expression in the afferent pathways and brainstem nuclei [22]. This may contribute to reduced hypertension risk in premenopausal females compared with males.

In terms of autonomic function, preclinical models of hypertension have also shown sex-dependent mechanisms extend to circadian profiles. Thus far, these studies often show an attenuated impact on physiological endpoints in females compared to males. Interestingly, angiotensin II-induced hypertension disrupts circadian rhythms in both sexes but produces distinct physiological consequences such as altered heart rate and activity rhythms in females, and reduced BRS in males [50]. Similarly, when circadian stress is combined with a high-fat diet in a Bmal1 KO rat model, males show greater BP elevation and a disruption of sodium homeostasis compared to females [12].

Circadian disruption studies also indicate sex differences in autonomic responses. In a study by Prabhat et al., dim light at night caused a loss of BP rhythms in both sexes [37]. However, this loss of amplitude was due to lower BP at night in females but higher BP in males during the day. This aligns with observed differences in autonomic function where females had reduced nocturnal sympathetic activity and males had increased daytime sympathetic activity [37]. Of note, time-restricted feeding was shown to normalize these changes suggesting that dietary interventions may be a therapeutic strategy to reduce the adverse effects of dim light at night.

Together, these studies suggest that sex has an influence on both the vulnerability to circadian disruption and the downstream expression of autonomic and cardiovascular phenotypes. Some of the key mechanistic questions that remain include the role of sex hormones in modulating clock gene function within autonomic nuclei, sex differences in SCN output signaling, and whether males and females differ in susceptibility to specific forms of circadian disruption. These gaps have important implications for studying whether chronotherapies may be sex specific.

## Conclusions

The interface between neural control of BP and circadian physiology represents a core principle of cardiovascular regulation that integrates endogenous timing systems with neural circuits that control BP. When this system is intact, it produces stable 24-hour blood pressure rhythms characterized by nocturnal dipping and a controlled morning surge. When disrupted, it gives rise to phenotypes such as non-dipping hypertension and exaggerated MBPS which are associated with increased cardiovascular disease risk. Despite advances in recent years, major gaps remain in defining the causal pathways linking circadian disruption to hypertension, and in understanding how sex differences shape neural control of 24-hour BP across the lifespan. Circadian rhythms do not just have a minor or occasional influence on cardiovascular systems, such as with shift work or jetlag, but are a fundamental component of cardiovascular health that impacts the risk of hypertension and related end organ disease. Developing strategies to restore and improve circadian disruption of biological systems offers a potential therapeutic strategy for mitigating hypertension.

## Perspectives and Future Directions

There is growing evidence that indicates circadian regulation is a key component of neural BP control. While there have been advances in identifying brain regions that are involved in autonomic regulation, the mechanisms by which molecular clocks within autonomic nuclei shape neuronal activity and sympathetic output remain unclear. Defining how circadian timing translates into changes in autonomic function will be important in understanding why disruptions in rhythms contribute to the development of hypertension.

Currently, there are still several challenges that need addressing. One such challenge is to establish the causal contribution of local molecular clocks within autonomic nuclei to BP regulation. Additionally, there needs to be more emphasis on the interactions between the central circadian clock and peripheral clocks in the kidney, vasculature, adrenal gland, and immune system. By understanding how these oscillators communicate, it may reveal why circadian disruptions produce various cardiovascular outcomes across individuals.

Emerging studies also suggest that neuroimmune signaling represents an important link between circadian dysfunction and sympathetic dysregulation. It still remains unclear whether microglial clock disruption is a common underlying mechanism in neurogenic hypertension. It is also unclear whether restoring proper circadian function reverses disease progression. In addition, promising evidence indicates sex-specific regulation of autonomic function and responses to circadian disruption. Future studies in humans and animal models need to incorporate both sexes and be mindful of the influence of hormonal status.

In summary, the integration of circadian biology with neurophysiology has the potential to redefine how the control of hypertension is understood and treated (Fig. 1). Therapeutic techniques such as ambulatory blood pressure monitoring, chronotyping, molecular phenotyping, and chronotherapy may target both the magnitude of BP reduction as well as restore cardiovascular rhythmicity.

## Key References

7. *Faraci FM, Scheer F. Hypertension: Causes and Consequences of Circadian Rhythms in Blood Pressure. Circ Res. 2024;134(6):810-32. https://doi.org/10.1161/CIRCRESAHA.124.323515.

○ Importance: This review provides a recent synthesis of the mechanisms linking circadian rhythms with BP regulation and hypertension and serves as an importance framework for the broader concepts discussed in this review.

12. **Latimer MN, Rhoads MK, Reynolds LD, Pollock DM. Chronic circadian stress impairs blood pressure and sodium homeostasis in a diet- and sex-specific manner. Am J Physiol Renal Physiol. 2026;330(2):F186-F98. https://doi.org/10.1152/ajprenal.00316.2025.

○ Importance: This study provides recent evidence that chronic circadian stress disrupts BP and sodium homeostasis in a sex- and diet-dependent manner, highlighting the interaction between circadian disruption and other cardiovascular risk factors.

14. **Zhang S, Zhao D, Huang Y, Chen M, Wang F, Yang S et al. SIRT1 targeted bioengineered extracellular vesicles chrono-reprogram microglia to reverse non-dipping hypertension - myocardial remodeling and comorbid neuropsychiatric disorders under stress. Int Immunopharmacol. 2025;166:115542. https://doi.org/10.1016/j.intimp.2025.115542.

○ Importance: This study provides mechanistic evidence linking impaired microglial clock function with non-dipping hypertension and demonstrates that restoring microglial circadian function can improve BP rhythmicity, supporting a role for neuroimmune mechanisms in circadian BP dysregulation

23. *Ragozzino FJ, Peterson BA, Karatsoreos IN, Peters JH. Circadian regulation of glutamate release pathways shapes synaptic throughput in the brainstem nucleus of the solitary tract (NTS). J Physiol. 2023;601(10):1881-96. https://doi.org/10.1113/JP284370.

○ Importance: This study provides mechanistic evidence that circadian rhythms regulate glutamatergic signaling in the NTS, supporting the concept that local circadian mechanisms can influence brainstem circuits involved in baroreflex and autonomic regulation.

37. **Prabhat A, Sami D, Ehlman A, Stumpf I, Seward T, Su W et al. Dim light at night unmasks sex-specific differences in circadian and autonomic regulation of cardiovascular physiology. Commun Biol. 2024;7(1):1191. https://doi.org/10.1038/s42003-024-06861-8.

○ Importance: This study demonstrates sex-specific effects of circadian disruption on BP rhythms and automatic regulation, providing direct evidence that males and females can respond differently to environmental circadian stress.

50. **Visniauskas B, Ogola BO, Kilanowski-Doroh I, Harris NR, Diaz ZT, Horton AC et al. Hypertension disrupts the vascular clock in both sexes. Am J Physiol Heart Circ Physiol. 2024;327(4):H765-H77. https://doi.org/10.1152/ajpheart.00131.2024.

○ Importance: This study demonstrates that hypertension is associated with disruption of cascular clock function and identifies sex-dependent changes in cardiovascular regulation, supporting the importance of local clocks and sex in circadian BP regulation.

61. **Pigazzani F, Dyar KA, Morant SV, Vetter C, Rogers A, Flynn RWV et al. Effect of timed dosing of usual antihypertensives according to patient chronotype on cardiovascular outcomes: the Chronotype sub-study cohort of the Treatment in Morning versus Evening (TIME) study. EClinicalMedicine. 2024;72:102633. https://doi.org/10.1016/j.eclinm.2024.102633.

○ Importance: This study provides clinical evidence that the relationship between antihypertensive medication timing and cardiovascular outcomes may depend on individual chronotype, supporting a more personalized approach to chronotherapy.

73. *Theodorakopoulou MP, Iatridi F, Karagiannidis AG, Georgiou A, Manti S, Karpetas A et al. Association of Dialysis Shift with Morning Surge in Blood Pressure and Dipping Pattern. Am J Hypertens. 2026;39(5):716-22. https://doi.org/10.1093/ajh/hpaf218.

○ Importance: This study demonstrates that the timing of a clinical treatment can influence BP dipping and morning surge, providing a recent example of how treatment timing may affect BP rhythmicity in a clinical population.

## Data Availability

No datasets were generated or analysed during the current study.
